# Temporal patterns in the social network of core units in Rwenzori Angolan colobus monkeys: Effects of food availability and interunit dispersal

**DOI:** 10.1002/ece3.7274

**Published:** 2021-03-05

**Authors:** Frances V. Adams, T. Jean M. Arseneau‐Robar, Tyler R. Bonnell, Samantha M. Stead, Julie A. Teichroeb

**Affiliations:** ^1^ Department of Anthropology University of Toronto Scarborough Toronto ON Canada; ^2^ Department of Psychology University of Lethbridge Lethbridge AB Canada

**Keywords:** colobines, core unit associations, food availability, food competition, male dispersal, social network analysis

## Abstract

1. Multi‐level societies are complex, nested social systems where basic social groups (i.e., core units) associate in a hierarchical manner, allowing animals to adjust their group sizes in response to variables such as food availability, predation, or conspecific threat. These pressures fluctuate over time and examining the extent to which this variation affects the clustering of core units into different tiers may be instrumental in understanding the evolution of multi‐level societies.
2. The goal of our study was to determine the degree of temporal variability in interunit associations in a multi‐level society of Rwenzori Angolan colobus monkey (*Colobus angolensis ruwenzorii*), and to determine the social and ecological factors that underlie association patterns. The *C. a. ruwenzorii* multi‐level society consists of at least three tiers, with core units clustering into clans that share a home range in a band tier.
3. We performed social network analyses on 21 months of association data from 13 core units (totaling 139 identifiable individuals) at Lake Nabugabo, Uganda. We described the patterns of variation in core‐unit associations over time and investigated how changes in rainfall, food availability, and interunit dispersals were correlated with these associations over the short‐term (month to month) and long‐term (year to year).
4. Although clans were relatively stable, larger‐scale changes in association patterns included the formation of an all‐male unit and the transfer of one core unit between clans (within the band tier). Seasonally, core units associated significantly more when fruit, their preferred food source, was abundant (i.e., social networks were denser and more clustered) and there was no direct effect of rainfall seasonality or young leaf availability. Male dispersals also occurred more during periods of high fruit availability, suggesting that greater band cohesion allowed males to prospect and transfer between core units. Once males transferred, their previous and new units associated significantly more with one another than with other core units for 1–2 months postdispersal. The dispersal of five males from one core unit to another in a different clan co‐occurred with this core unit switching its clan affiliation.
5. By examining temporal shifts in social network structure among core units, this study shows the interconnected roles that food availability and dispersal have in shaping the *C. a. ruwenzorii* multi‐level social system. Our findings highlight how ecological conditions can drive association patterns, impact interunit relationships, and influence social organization.

## Graphical abstract

Core units in a multi‐level society of Rwenzori Angolan colobus monkeys show temporal variation in association that corresponds to food availability. Units are more clustered when fruit is abundant and interunit dispersal by males also peaks at this time, suggesting that the seasonal pattern of band cohesion allowed males to prospect for dispersal opportunities. Our findings highlight how ecological conditions can influence association patterns, interunit relationships, and ultimately social organization.

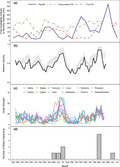

## 1 INTRODUCTION

Complex, hierarchical social systems, termed multi‐level societies, are present in species from many distantly related taxa, such as birds (Papageorgiou et al., 2019), cetaceans (Whitehead et al., 2012), equids (Rubenstein & Hack, 2004), proboscideans (Wittemyer et al., 2005), primates (Grueter et al., 2012), and chiropterans (Kerth et al., 2011). Determining why these types of societies evolve and how they function are key questions in biology. In multi‐level societies, stable subgroups (hereafter core units) associate in a hierarchical manner, clustering into successive levels or tiers (Grueter et al., 2012; Grueter et al., 2017). One to four tiers of nonrandom association have been documented, with higher tiers numbering hundreds of individuals in some species (Grueter, Matsuda, et al., 2012; Schreier & Swedell, 2012a; Snyder‐Mackler et al., 2012; Wittemeyer et al., 2005). The factors that determine the number of tiers and their composition(s), as well as the ways that ecological and social pressures affect their stability are still poorly understood for most species that form multi‐level societies (Farine et al., 2015; Grueter et al., 2017).

Multi‐level social organizations appear to have evolved because they allow animals to adjust group size more fluidly than is possible in stable groups (Aureli et al., 2008; Grueter et al., 2017, 2020). The advantages and disadvantages of group living have been well documented (Krause & Ruxton, 2002). Large aggregations are beneficial, primarily because of the multiple ways that they lower predation risk (i.e., detection, dilution, predator confusion, defence, Hamilton, 1971; Pulliam & Caraco, 1984), while the chief cost of large group size is the increase in food competition that results from many conspecifics together (Terborgh & Janson, 1986). Indeed, there are examples of multi‐level societies forming large aggregations at higher tiers when predators are nearby (e.g., *Papio hamadryas*, Schreier & Swedell, 2012b; *Physeter microcephalus*, Whitehead et al., 2012), and fissioning to lower tiers when resource availability is reduced (e.g., *Loxodonta africana*, Wittemyer et al., 2005; *Orcinus orca,* Foster et al., 2012; *Papio hamadryas*, Schreier & Swedell, 2012b; *Rhinopithecus roxellana*, Qi et al., 2014). There can be important social advantages to aggregation as well. For instance, mates are readily available and can be monitored (Krause & Ruxton, 2002; Wrangham, 1979), and individuals can form coalitions to defend mates (Grueter & van Schaik, 2009; Pappano et al., 2012; Rubenstein, 1986; Rubenstein & Hack, 2004; Xiang et al., 2014) or food (Cheney & Seyfarth, 1987; Wrangham, 1980) from conspecifics. Pooling of information in larger groups may also lead to more accurate navigational accuracy (Cantor et al., 2020; Couzin et al., 2011), although there may be limits to this advantage because of the consensus costs and the constraints of moving as a large group (Papageorgiou & Farine, 2020).

Many of the ecological and social pressures that determine how beneficial or costly aggregation is show temporal fluctuations, making the flexibility inherent in multi‐level societies particularly advantageous. For example, predator movements and prey‐switching can change predation risk over time (e.g., Metz et al., 2012), dry seasons may lead to aggregations where surface water remains available (e.g., Chamaillé‐Jammes et al., 2008; Valeix, 2011), food availability and distribution fluctuate following seasonal shifts in rainfall (e.g., Schradin & Pillay, 2006; Takemoto, 2004), and breeding seasonality may drive temporal changes in aggregation (e.g., Baden et al., 2016; Dudgeon et al., 2008). When conditions allow larger aggregations to form and individuals or core units move into closer proximity, they have greater opportunities to observe one another and to interact. Thus, this temporal clustering allows individuals to assess mating and dispersal opportunities in other groups or core units (Clobert et al., 2009; Mares et al., 2014), which could potentially lead to a seasonal pattern in dispersals (e.g., Ekernas & Cords, 2007; Yao et al., 2011; Young et al., 2019).

**FIGURE 1:**
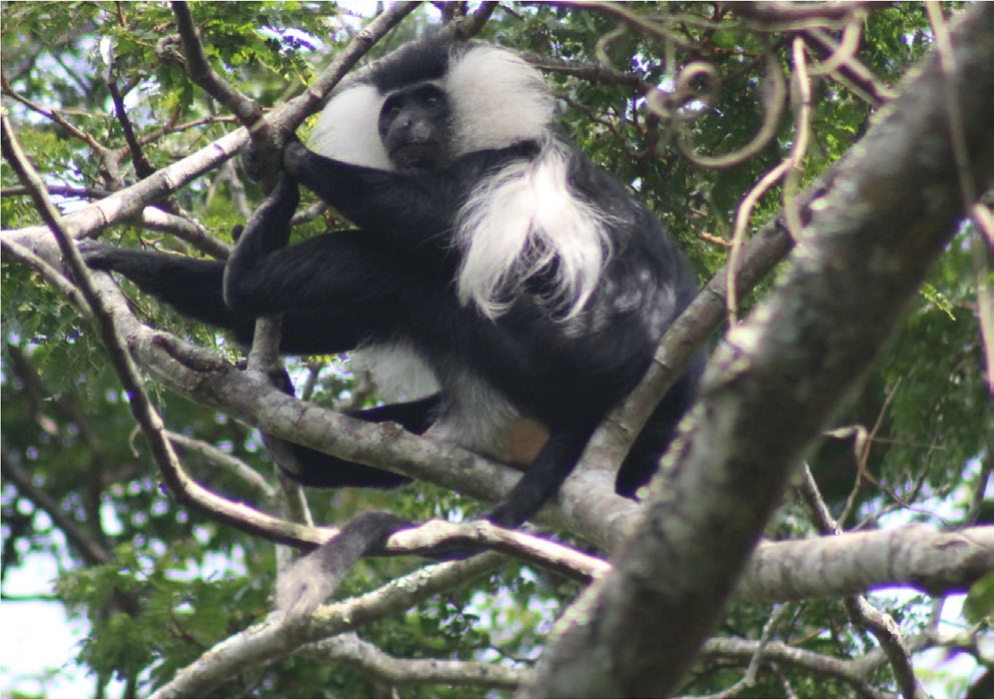
An adult male *Colobus angolensis ruwenzorii* at Nabugabo, Uganda. (Photo credit: Samantha Stead)

Our goals were (a) to determine the degree of temporal variability in interunit associations and clan stability in a recently discovered multi‐level society of Rwenzori Angolan colobus monkey (*Colobus angolensis ruwenzorii*) (Figure 1); (b) to examine whether changes in rainfall and/or food availability influenced temporal changes in association patterns; and (c) to assess whether changes in clustering led to seasonal peaks in dispersals between core units. We did not investigate the effect of predation on temporal aggregation patterns because our study population at Lake Nabugabo, Uganda occurs within a series of forest fragments where many natural predators are extirpated and local people do not hunt primates. The main predation risk is from people's dogs (*Canis familiaris*, Adams & Teichroeb, 2020); a pressure that is unlikely to be seasonal in nature. The multi‐level society that *C. a. ruwenzorii* form is unique among primates in that it contains not only one‐male/multi‐female units (OMUs), but also core units that are multi‐male/multi‐female (MMUs) (Miller et al., 2020; Stead & Teichroeb, 2019) with up to eight socially integrated, reproductive males (Stead & Teichroeb, 2019). There are at least three tiers of social organization. Core units fission and fuse with one another throughout the day but associate preferentially with core units from the same clan. Clans share a home range in a band tier of organization. Initial cluster analyses with one year of data revealed two clans in our study band of 12 core units (Stead & Teichroeb, 2019). Preliminary data shows that though both males and females disperse from their natal core unit in *C. a. ruwenzorii*, males transfer into other core units within the band while most females observed to disperse (3/4, 75%) have emigrated out of the band (Stead & Teichroeb, 2019).

Using core unit associations observed over 21 months at Nabugabo, we first examined whether clan‐level groupings of *C. a. ruwenzorii* core units stayed the same over time using hierarchical cluster analyses. Second, we examined temporal variation in core unit clustering using social network analysis relative to ecological (seasonality in rainfall and food availability) and social (interunit dispersals) conditions. We hypothesized that food availability and interunit dispersals would influence clan stability and the degree of clustering among *C. a. ruwenzorii* core units over time, but we did not expect a direct relationship with rainfall patterns. Water availability is important for primates and arboreal colobus monkeys occasionally drink from water sources like tree cavities (Nowak, 2008; Teichroeb et al., 2009). However, primates can also obtain water from succulent foods like young leaves and fruits (Altmann, 1998) and can even lick dew off of foliage (Onderstepoort, 1988). We did not expect these sources of water to have a clumped distribution during the dry seasons in the forest at Nabugabo, and thus we did not predict that they would influence patterns of aggregation among core units. Greater overall food availability should allow larger aggregations to form because food competition is alleviated (e.g., Foster et al., 2012; Schreier & Swedell, 2012b; Wittemyer et al., 2005). Young leaves make up the majority of the annual diet (65%) of *C. a. ruwenzorii* at Nabugabo but fruits are the only food type positively selected for and these are a high‐quality resource (2021). We therefore predicted that: (a) core unit clustering would be influenced most strongly by the availability of fruits, and that core units would associate more during times of high fruit availability. This temporal clustering of groups due to resource availability should allow individuals greater opportunities to assess dispersal opportunities in other core units (e.g., Clobert et al., 2009; Mares et al., 2014). Thus, we further predicted that high association rates among core units at times of peak fruit availability would: (b) lead to a seasonal pattern of male dispersal between core units. In primates, it often takes time for bonds among former group members to sever and to establish bonds in new groups (Isbell & Van Vuren, 1996). Therefore, we predicted that: (c) these dispersals would change clan make‐up when core units that recently had males disperse between them were from different clans (i.e., this would lead to greater association between core units from different clans during dispersal periods compared to when no transfers are occurring, potentially altering long‐term core unit associations).

## 2 METHODS

### 2.1 Study species and site

We conducted this study on a population of wild Rwenzori Angolan colobus monkeys (*Colobus angolensis ruwenzorii*) (aka Adolf Friedrich's Angolan colobus) located in a forest fragment near Lake Nabugabo, Masaka District, central Uganda (0°22′‐12°S and 31°54′E). Lake Nabugabo is a small lake (8.2 × 5 km) west of Lake Victoria surrounded by a mix of swamp, wetland, grassland, primary and secondary forest, and degraded patches of forest (Chapman et al., 2016). This study focused on a band (TR band) of 132–139 colobus in 12–13 core units including one all‐male unit (Stead & Teichroeb, 2019), occupying a moist tropical forest fragment made up partly of the Manwa Forest Reserve (~280 ha) lying on the west side of Lake Nabugabo (Teichroeb et al., 2019). The forests that the study band occupies are at a mean elevation of 1,151 m with a relatively flat terrain (range: 1,134–1,167 m). Annual rainfall in this area during the August 2017‐ July 2018 period was 758.59 mm across two rainy seasons, one from February to May and another from September to November. The mean annual temperature was 22.2°C (min. 18.7°C, max. 26.2°C) (Adams & Teichroeb, 2020). The three most dominant tree species in the forest, in terms of both stem number and basal area, are *Pseudospondias microcarpa* (Anacardiaceae), *Maesopsis eminii* (Rhamnaceae), and *Funtumia latifolia* (Apocynaceae) (Teichroeb et al., 2019).

### 2.2 Data collection

Core unit associations were recorded during behavioral follows conducted over 21 months between August 28th, 2017 and May 13th, 2019 (243 days) by two trained field assistants (E. Mujjuzi and H. Kakeeto). The 21 continuous months of data collection covered four rainy seasons. From August 28th, 2017 to August 22nd, 2018, 12 core units were sampled and from August 29th, 2018 to May 13th, 2019, 13 units were sampled because an all‐male unit (AMU) had formed by the splitting off of seven adult males from the largest core unit (Lovoa), which subsequently became an OMU. One focal unit was followed each day between 7:00 hr −16:00 hr and all individuals were identified based on physical characteristics (e.g., broken fingers, tail shape, nipple coloration). Scan samples on core unit association were taken every two hours, where the number and identity of core units within a 50 m radius of the focal core unit was recorded along with the time and date (overall *N = *907 scans). We chose a two‐hour interval between scans to ensure their independence. We reasoned that two hours was enough time for core units to shift their position relative to one another (Stead & Teichroeb, 2019). Our data collection regime led to a relatively even distribution of focal days among core units during the study (mean *N* days/unit = 20.17, range: 14–25; mean *N* scans/unit = 75.25, range: 56–90). Dispersals of individuals within the study band were recorded on notice of occurrence. We then generated a date range during which the dispersal occurred based on the last time an individual was noted in their original core unit. The month of dispersal was determined to be the month with the most potential dates within that range.

To examine the seasonality of association patterns, we considered three ecological variables: rainfall, the availability of young leaves, and the availability of fruits. Rainfall data (mm per month) was obtained from https://www.worldweatheronline.com/masaka‐weather‐history/masaka/ug.aspx for the nearby town of Masaka (12.5 km away). We considered the availability of young leaves and fruits as these food items comprise the majority of the *C. a. ruwenzorii* diet at this field site (96%, 2021). Food availability indices were calculated for each of these plant parts, for each month of the study period. We used a line‐transect survey to estimate tree species abundance (i.e., number of trees and their basal area) within the home range of the *C. a. ruwenzorii* band. Thirty‐two parallel transects set 100 m apart were cut throughout a 140 ha section of the forest and all trees >10 cm DBH within 5 m of either side of the transect were identified and measured (covering 9.702 ha) (Teichroeb et al., 2019). The seasonal availability of these plant parts was estimated using monthly phenology surveys of 126 trees of 44 species that were known to be consumed by *C. a. ruwenzorii*. During phenology surveys, observers indexed the percent canopy cover of mature versus young leaves, ripe and unripe fruit, ripe and unripe seed pods, and buds versus flowers with a sample of three trees of most species. The proportion of the crown covered in each plant part was assessed on a five‐point scale (0 = plant part not present, 1 = 1%–25% covered, 2 = 26%–50% covered, 3 = 51%–75% covered, and 4 = 76%–100% covered). We calculated the food availability index for both young leaves and fruits separately by multiplying the mean monthly phenology score for each plant part in each of the 44 species by the total basal area of that species, and summing these values for all the tree species consumed (Dasilva, 1994; Fashing, 2001; Saj & Sicotte, 2007). These methods have previously been shown to capture fruiting and leafing peaks in line with the colobus dietary choices (2021).

### 2.3 Cluster analyses and preferred associations

We used a hierarchical cluster analysis run with SOCPROG (v.2.9: Whitehead, 2009) to determine if the clustering of core units into clans was consistent over time. This method performs agglomerative clustering of groups or individuals based on their similarities, which in this case was a dyadic value of association. To examine larger‐scale changes in clan affiliation for core units, we split the data into two sampling periods to compare metrics by year: August 28th, 2017 – August 22nd, 2018 (sample period 1) and August 29th, 2018 – May 13th, 2019 (sample period 2). We created an association matrix for each year by calculating the simple association index of each dyad, where a value of 1 indicates the two core units were always in association in the sample and 0 indicates they were never in association in the sample. The simple association index (AI) was chosen because we could positively identify all core units in association with the focal unit (Whitehead, 2008). AI was calculated as AI = *N*_AB_/(*N*_A_ + *N*_B_) or the number of times that two core units were in association during scans, divided by the total number of scans where either unit was present. We examined the fit of our data with four different clustering methods (average linkage, Ward's weighted, complete linkage, and single linkage); these linkage criteria determine the distance between sets of observations as a function of the pairwise distances between observations (Whitehead, 2008). The average linkage method had the highest cophenetic correlation coefficient (CCC = 0.891) and thus the best fit with the data, so this was the clustering method that we used for hierarchical cluster analyses (see Stead & Teichroeb, 2019). We used dendrograms (Figure 3) created through the average linkage method to compare clustering into clans between sample periods. We followed Stead and Teichroeb (2019) and used a cutoff AI of 0.05 to define clan associations. We then conducted permutation tests for preferred/avoided associations using SOCPROG, and permuted association matrices 10,000 times to stabilize *p*‐values. Although these tests reveal large‐scale changes in clan composition, the results do not assess variability in clustering at smaller time‐scales. Thus, further analyses using smaller time windows was performed to adjust for this.

### 2.4 Smaller time‐window comparisons

To test the effects of our ecological and social variables on core unit social networks, we created 20 time‐aggregated networks using a 31‐day window size and a 31‐day window shift that spanned the full dataset (August 28th, 2017 to May 13th, 2019) using the R package netTS (Bonnell & Vilette, 2019) and visualized with the igraph package (Csardi & Nepusz, 2006). This program allows the user to alter the window size, dependent on the types of questions being asked. A benefit of using a shifting‐window is the ability to see patterns and variation that may not be detected in larger window size comparisons. We determined the optimal window size, in terms of maximizing variability in edge density for our data (31 days), using a bootstrap technique (supplementary material, Figure S1). Within each time‐window, a new network was created and we analyzed social network metrics to determine the connectedness of core units over time. At the node (core‐unit) level, we calculated degree (i.e., the number of core units associated with) and strength (i.e., sum of all edge weights for a given node, indicating the total association rate for a given core unit). At the network level, we calculated edge density (i.e., the ratio of the number of edges and the number of possible edges), clustering coefficient (i.e., the number of core units associated with that also associated with one another), and cosine similarity (see below). At the dyad level, we calculated dyad association measures (AI).

### 2.5 Statistical analyses

To analyze the likeness of the core unit social network over time, we used the cosine similarity metric. Cosine similarity measures the similarity of associations between two networks and can take into account the weight and presence of associations (Newman, 2010). A cosine similarity of 1 indicates that two networks are exactly the same, while a cosine similarity of 0 indicates that they do not have any shared associations (Newman, 2010). For this study, cosine similarity was used to measure the changes in associations from one time window to the next (Bonnell & Vilette, 2019). We compared cosine similarity for each core unit to the previous time window to reveal short‐term variability, as well as between each window and the first time window in the data set to reveal any long‐term variability. To assess uncertainty in our estimates of cosine similarity we repeated the same analysis on 100 bootstrapped samples of the observed data.

To examine the effects of ecological conditions on group clustering and the overall connectedness of core units, we modeled how changes in fruit and young leaf abundance and rainfall influenced both node and network level measurements (BRMS package; Bürkner, 2017). These ecological variables were only moderately correlated (highest *r* = 0.36) so multicollinearity and variation inflation, which generally occurs when *r* > 0.7 (Dormann et al., 2013), was not an issue. For network‐level measures (i.e., density and clustering coefficient at the band level), we used a linear regression with fruit availability, young leaf availability, and rainfall as predictors, and included AR1 autocorrelated errors. While for node‐level measures (i.e., strength and degree at the core unit level), we used a multi‐level model with fruit availability, young leaf availability, and rainfall as predictors, and core‐unit identity as a random effect, since nodes are repeatedly measured over time. For this model, we also included AR1 autocorrelated errors to account for the serial dependence in the network measures over time. In these models, we standardized all predictor variables and calculated r‐squared (*R* ^2^) values to provide estimates of effect size for each model (Gelman et al., 2019). In these models, we chose to use weakly informative priors centered on zero for all slopes, that is, normal(0,1), starting the model off with the highest probability at zero for all slopes. This approach has the advantage of reducing problems of multicollinearity between predictors and of starting the model off assuming no effects of our predictors. All models converged with rhats < 1.01 and effective sample sizes above 300. The results of postnetwork permutations (Figure S2, Table S2), and posterior predictive checks for each model are provided in the supplementary (Figures S3–S5) to aid in model interpretation.

To investigate the association between male dispersals and association patterns, we first determined whether there was temporal variability in male dispersal events. We examined the relationship between our ecological variables (rainfall, fruit availability, and young leaf availability) and the number of males transferring between core units in a given month using a Spearman rank correlation (coin package; Hothorn et al., 2006), applying a Bonferroni correction for multiple comparisons (α = 0.017). For these tests, we calculated Monte‐Carlo approximated *p*‐values as these are more robust when there are ties in the data (Hájek et al., 1999), which occurred because there were multiple months in which no males dispersed. Finally, we tested whether two core units were more likely to continue to associate after male(s) transferred between them than would be expected from baseline association levels. Here, we calculated a simple association measure (AI) between the core unit dyads with male transfers for each month (up to a maximum of three months) following the male dispersal event. For comparison, we calculated the baseline AI level between each of the core units involved in the dispersal event and other core units they had each been associated with during the month the male(s) transferred. We used one‐sample Wilcoxon signed‐ranks tests to determine if the association index between the two core units involved in male transfers was higher than their association indices with other core units (rcompanion package; Mangiafico, 2020). All analyses were done using either SOCPROG v.2.9 (Whitehead, 2009) and R v.3.6 (R Core Team, 2019).

## 3 RESULTS

### 3.1 A dynamic network

**FIGURE 2:**
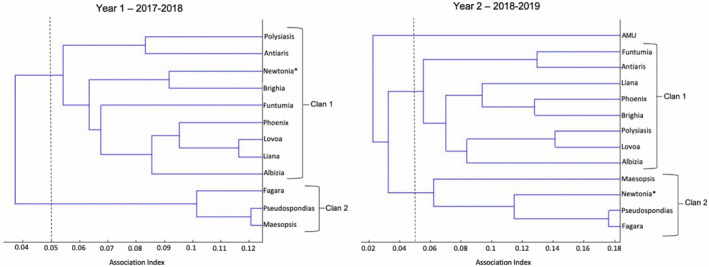
Comparison of *Colobus angolensis ruwenzorii* core units’ clan associations across years at Nabugabo, Uganda, depicted by dendrograms created using hierarchical cluster analysis (CCC = 0.891; SOCPROG: Whitehead, 2009). * indicates the movement of a core unit between clans. The “Year 1 – 2017–2018” dendrogram has been adapted from figure 1a in Stead and Teichroeb (2019)

Throughout the study, core unit compositions remained relatively stable with a total of nine males and one female dispersing between units within the band over the 21‐month study period in six dispersal events (i.e., two dispersal events involved the parallel transfer of males). Core unit associations varied over time, and clan composition changed from sample period one to two. Hierarchical cluster analysis showed that, compared to period one, two clans were still evident in period two but one core unit (Newtonia) had switched association between clans. In addition, the formation of the AMU, led to this unit forming its own branch in loose association with the two main clans (Figure 2). The results of the permutation tests for preferred relationships between core units showed significance for sample period one, and not for sample period two (Sample period one: CV_Obs_ = 0.484, CV_Rand_ = 0.44, *p* =0.014; Sample period two: CV_Obs_ = 0.150, CV_Rand_ = 0.150, *p =*0.8487). This suggests that core units demonstrated less preference when associating with other units in sample period two. Nonetheless, network metrics between sample period one and two were largely similar with little change in the averages for affinity, strength, centrality (Table S1). Most notably, we saw a decrease in the clustering of units between the two years (sample period 1: CC = 0.48, sample period 2: CC = 0.31).

**FIGURE 3:**
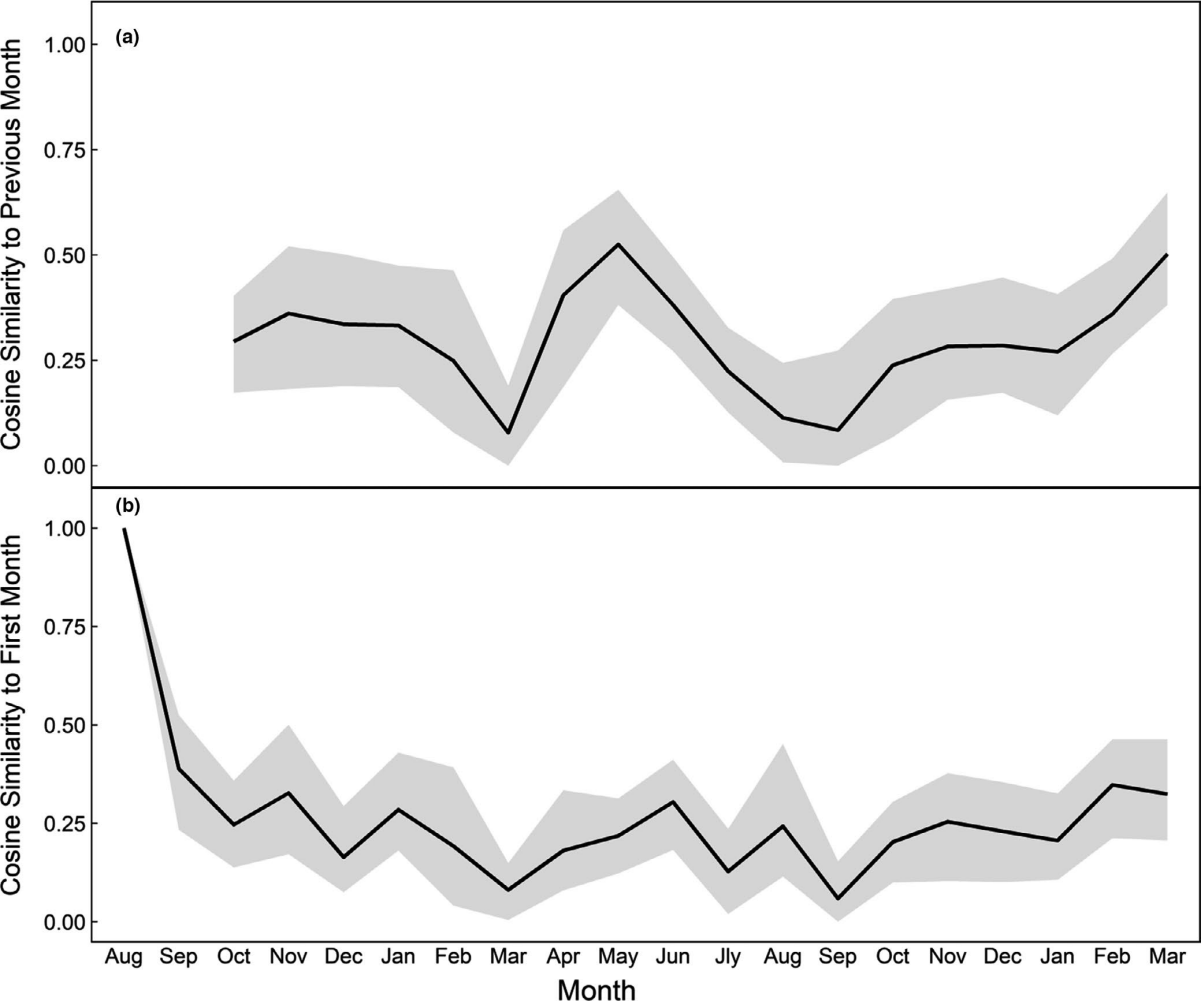
Stability of the social network between core units of *Colobus angolensis ruwenzorii* at Nabugabo, Uganda from August 2017 to May 2019 in both the (a) short‐term and (b) long‐term, as illustrated by the cosine similarity (a) to the previous month and (b) to the first month of the study period. Shaded areas indicate the 95% highest density interval from 100 bootstrap samples of the observed data

When looking at shorter sample windows, cosine similarity values demonstrate the high amounts of temporal variability within the combined sample years (2017–2019), both in the short‐term (Figure 3a) and the long‐term (Figure 3b). When comparing each window to the first (Figure 3b), cosine similarity ranges between ~0.12–0.51 (excluding the first month where cs = 1.0). The highest value of 0.51 occurred in the months of October and December 2017, and the lowest of 0.12 in April 2018. When comparing each window to the previous (Figure 3a), cosine similarity ranges between ~0.15–0.61. The highest value of 0.61 was seen in the month of April 2019, and the lowest of 0.15 in November 2018.

### 3.2 Ecological conditions and association patterns

**FIGURE 4:**
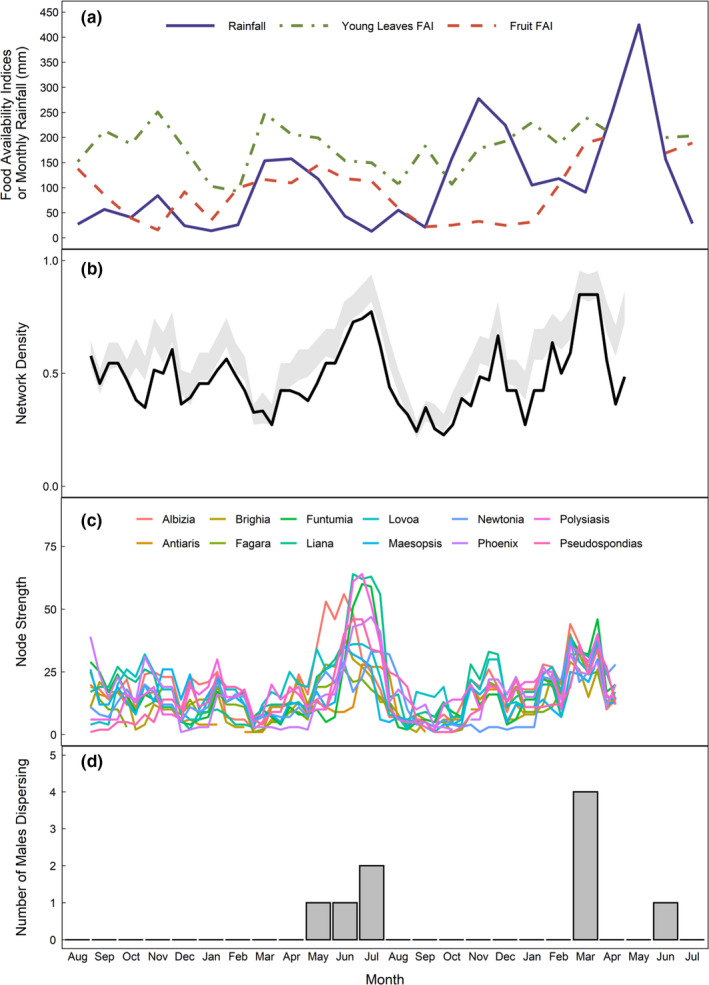
(a) Available data on ecological conditions (rainfall, young leaf and fruit availability) with (b) social network density measures, (c) node strength, and (d) the timing of male dispersal events from August 2017 to July 2019 in a band of *Colobus angolensis ruwenzorii* at Nabugabo, Uganda. Core unit association levels are represented by (b) and (c). For (b) the shaded area represents the density range expected due to chance encounters, and was calculated using data‐stream permutations using 95% CI

Neither rainfall, nor young leaf availability were strongly associated with network measures, either at the network level or the node level (Tables 1 and 2; Figure 4). However, association patterns did correlate with fruit availability. At the network level, fruit availability was weakly but positively associated with both network density and clustering coefficient (Table 1; Figure 4). At the node‐level, fruit availability showed a strong positive relationship with both strength and degree (Table 2; Figure 4). Thus, core units were more likely to associate with one another, and to form larger aggregations when fruits were abundant, but decreased associations when fruit was scarce (Figure 4a).

**TABLE 1:** Impact that ecological variables (i.e., food availability and rainfall) had on social network measures (i.e., density and clustering coefficient) of *Colobus angolensis ruwenzorii* core units at Nabugabo, Uganda at the network level from August 2017 to May 2019. Note that in the model, all ecological variables were scaled

	Estimate	Error	Lower 95% CI	Upper 95% CI
Density				
Intercept	0.54	0.06	0.44	0.65
Fruit availability	0.08	0.04	0.00	0.15
Young leaf availability	−0.02	0.03	−0.08	0.03
Rainfall	−0.02	0.03	−0.09	0.05
Clustering coefficient				
Intercept	0.63	0.04	0.54	0.72
Fruit availability	0.07	0.03	0.01	0.14
Young leaf availability	0.00	0.03	−0.06	0.04
Rainfall	−0.03	0.03	−0.08	0.03

Notes

Variance explained by models: footnote: density *R* ^2^ = 0.27 (0.04, 0.49), clustering coefficient *R* ^2^ = 0.28 (0.05, 0.50)

**TABLE 2:** Impact that ecological variables (i.e., food availability and rainfall) had on social network measures (i.e., strength and degree) of *Colobus angolensis ruwenzorii* core‐units at Nabugabo, Uganda at the node level from August 2017 to May 2019. Note that in the model, all ecological variables were scaled

	Estimate	Error	Lower 95% CI	Upper 95% CI
Strength				
Intercept	15.95	1.04	13.90	17.89
Fruit availability	2.23	0.68	0.81	3.49
Young leaf availability	−0.89	0.46	−1.78	0.02
Rainfall	−0.85	0.56	−1.89	0.31
Degree				
Intercept	5.93	0.24	5.49	6.41
Fruit availability	0.94	0.17	0.49	1.18
Young leaf availability	−0.23	0.14	−0.50	0.05
Rainfall	−0.23	0.16	−0.55	0.09

Note

Variance explained by model's footnote: strength *R* ^2^ = 0.08 (0.04, 0.15), degree *R* ^2^ = 0.14 (0.07, 0.22)

### 3.3 Male dispersal and association patterns

**FIGURE 5:**
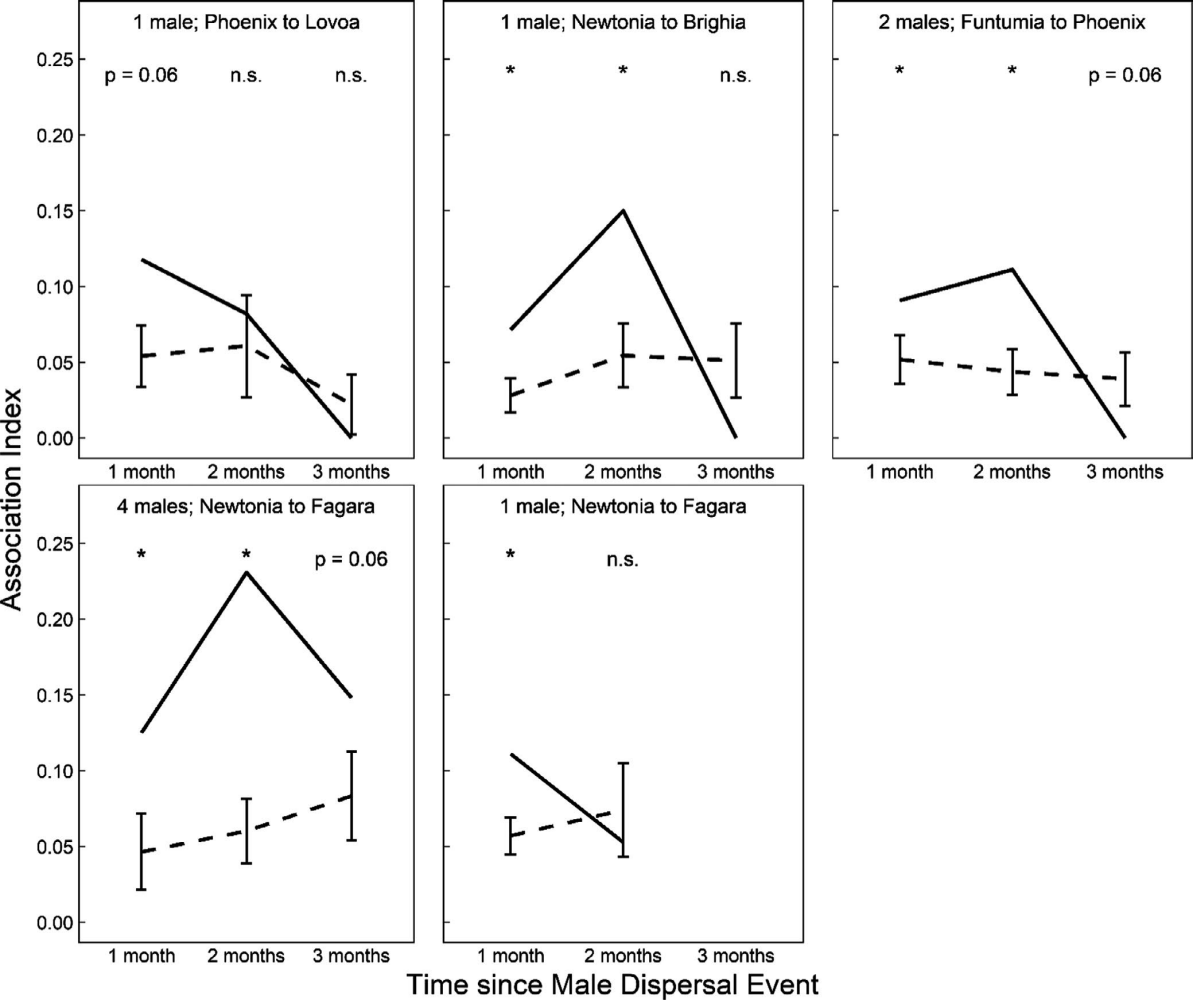
Association indices for the *Colobus angolensis ruwenzorii* core units at Nabugabo, Uganda that males transferred between (solid lines) for the 3 months following each male dispersal event from August 2017 to May 2019. Dashed lines represent expected (baseline) levels of association, given how much these core units (i.e., those involved in male transfers) continued to associate with other core units not involved in the male dispersal event. (*) indicates association indices that were significantly higher in the core units with male transfer than would be expected at α = 0.05

There was a significant correlation between the availability of fruits and the number of males transferring between core units in a given month (Spearman: *Rho* = 0.50, *Z* = 2.33, Monte‐Carlo approximated *p*‐value = 0.048). Conversely, there was no relationship between the number of male transfers and rainfall (*Rho* = 0.14, *Z* = 0.71, *p* = 1.00), or the availability of young leaves (*Rho* = 0.22, *Z* = 1.05, *p* = 0.87). Thus, male transfers were most likely to occur when fruits were abundant and core units aggregated. Furthermore, the core units in which males transferred between were more likely to keep associating (i.e., maintaining a high AI) than would be expected given their baseline level of association with other units. Analyses revealed significantly higher associations (*p* <0.05) of the dispersal dyad for 1–2 months postdispersal than would be expected given their association with control core units (Figure 5). However, we found that by the third postdispersal month, all dispersal dyad AIs were no longer significantly different from the baseline. It is noteworthy that the core unit that switched its clan association from clan 1 to clan 2 between the two sampling periods (Newtonia) may have had this increase in association with clan 2 because five males dispersed from this core unit to a unit in clan 2 (Fagara).

## 4 DISCUSSION

Our analyses revealed a dynamic social network between core units in the *C. a. ruwenzorii* multi‐level society. As we predicted, the clan tier of organization was not entirely stable over time. We observed two major changes in our study band. First, an all‐male unit formed when seven males left the largest core unit and began to range in loose association with the two clans. Second, one core unit moved between clans after dispersal events involving five males. These changes show that clans do shift in core unit composition over time, though not frequently, and that male dispersals can influence this variation.

Most core units maintained their clan associations from period one to period two, which could be due to underlying between‐unit kinship or familiarity, particularly among the band‐philopatric males (Stead & Teichroeb, 2019). Unlike many other social orders of mammals, primates tend to form stable groups over relatively long periods of time that are structured by the bonding of the philopatric sex (Altmann et al., 1996; Di Fiore, 2012; Silk, 2001, 2002). These kin‐based systems often extend beyond the smallest social unit to higher tiers in primate multi‐level societies (*Papio hamadryas, Theropithecus gelada*, Colmenares, 2004; Snyder‐Mackler et al., 2014; *Gorilla gorilla*, Morrison et al., 2019). We do not yet have kinship data for our study population but although both males and females disperse from their natal core unit in *C. a. ruwenzorii*, males transfer into other core units within the band while most females emigrate out of the band (Stead & Teichroeb, 2019). Thus, male kinship could structure relations between core units, leading to relatively stable clans over time.

We found support for our predictions regarding how ecological and social variables affect the degree of association among core units. Temporal changes in rainfall were not directly correlated with the amount of core unit clustering. Association patterns fluctuated at both the node and network level, with the largest changes correlating to seasonal shifts in fruit availability. As predicted, core units were more likely to associate, and did so with a larger number of other core units, when fruits, a preferred resource for this population (2021), were abundant. This increase in association was correlated with the timing of male dispersals between core units in the band. Our analysis of association indices following each male dispersal event within the band revealed that male transfers promote higher than expected dyadic associations between interacting core units in the short‐term (1–2 months postdispersal).

Many species alter their behavior in response to changing resource availability (Candolin & Wong, 2012) and our results show that *C. a. ruwenzorii* is no exception. Similar to studies done on other primates (*Cercocebus torquatus,* Dolado et al., 2016; *Rhinopithecus bieti,* Ren et al., 2012; *Papio hamadryas*, Schreier & Swedell, 2012b; *Pongo pygmaeus,* Sugardjito et al., 1987) and nonprimates (*Orcinus orca,* Foster et al., 2012; *Loxodonta africana*, Wittemyer et al., 2005), we found that *C. a. ruwenzorii* units increase their association levels during times of peak food availability. Food competition decreases when resources are abundant, allowing animals to aggregate if they choose, which allows them to take advantage of the benefits that large groups have for predator avoidance (Hamilton, 1971; Sueur et al., 2011). Species living in a multi‐level society benefit from this ability to alter overall group size in response to external pressures (Grueter & van Schaik, 2009). For *C. a. ruwenzorii*, enlarged group size may even mean an expansion of the microhabitats they are willing to take advantage of. Adams and Teichroeb (2020) found that at Nabugabo, where predation risk is greatest near the ground, *C. a. ruwenzorii* were willing to come lower in the canopy to find food when more core units were clustered together and predation risk was lessened. The analyses presented here suggest that this niche expansion may occur more often in resource rich seasons when core units are able aggregate.

Although we find correlations between seasonal fruit availability, association patterns and male dispersal, it is important to acknowledge that we cannot determine cause and effect between these phenomena. While we posit that higher fruit availability leads to more clustering among core units, which facilitates male dispersal, it is possible that males prospect more during seasons of food abundance and that male prospecting behavior drives the observed changes in association patterns. Seasonal dispersal patterns are found in many species (Stenseth & Lidicker, 1992) but in most cases, this pattern emerges due to seasonal breeding (e.g., *Presbytis entellus,* Borries, 2000; *Suricata suricatta*, Mares et al., 2014; *Chlorocebus pygerythrus,* Young et al., 2019; *Rhinopithecus roxellana,* Yao et al., 2011). Breeding is not typically seasonal in black‐and‐white colobus monkeys (Fashing, 2011), and we do not have data showing seasonal breeding at Nabugabo. Alternatively, it is sometimes advantageous for animals to time dispersal to coincide with high food availability because it allows them to compensate for increased travel, potentially in unfamiliar areas (Isbell & Van Vuren, 1996; Pusey & Packer, 1987). This explanation is unlikely to apply in a multi‐level society like that seen in *C. a. ruwenzorii*, as all the core units in our band share a home range (Stead & Teichroeb, 2019). Consequently, male dispersal between units does not require extra travel or moving into a new, unfamiliar area. We suggest that the best explanation for the seasonal pattern of male dispersal that we observe in *C. a. ruwenzorii* is the opportunity for prospecting provided by greater core unit clustering due to high resource availability. The proximity of so many other core units allows males to assess their composition (i.e., sex ratio) as well as the competitive ability of the males there (Teichroeb et al., 2020), potentially influencing their decision to disperse. In primates, it is common for dispersal to occur during intergroup encounters (e.g., *Macaca mulatta*, Boelkins & Wilson, 1972; *Erythrocebus patas*, Rogers & Chism, 2009; *Gorilla beringei*, Sicotte, 1993; *Rhinopithecus roxellana*, Yao et al., 2011) or to groups where prospecting has previously been directed (e.g., *Colobus vellerosus*, Teichroeb et al., 2011).

The persistence of high association indices postdispersal for core units that have males transfer between them may be a result of the continued bonds between individuals that persist even after the dispersal has taken place. The dispersing individual(s) likely still have ties in their former (sometimes natal) unit, which may contain many kin. However, over time, we see a slow decrease of association between the units individual(s) dispersed to and from, back to the baseline association levels that they have with other core units. This decrease in association may be explained by the further integration of the dispersing individual(s) into their new unit, and/or the seasonal decrease in fruit availability, and subsequent increase in food competition. Although, one large transfer event of five males between Newtonia and Fagara core units, led to Newtonia switching clan association. Future research examining how male‐male genetic and social relationships impact association patterns over short and long time periods will provide insights into the ways that kinship structures core unit association in tandem with ecological and social factors (e.g., Snyder‐Mackler et al., 2014).

To conclude, our results show that in the dynamic social network of Rwenzori Angolan colobus monkeys, core units behaviorally adapt to changing ecological conditions by altering their association patterns. Doing so has cascading effects on the composition of core units, and structure of both the clan and band tiers in this multi‐level society. This type of behavioral flexibility allows animals to thrive in dynamic environments (Candolin & Wong, 2012; Grueter et al., 2020). Our study provides a deeper understanding of the mechanisms underlying the formation of complex multi‐level social organizations and some insight into the intertwined temporal effects of ecological and social variables.

## CONFLICT OF INTEREST

There are no conflicts of interest to declare.

## AUTHOR CONTRIBUTION

**Frances V. Adams:** Conceptualization (equal); Formal analysis (equal); Writing‐original draft (lead); Writing‐review & editing (equal). **T. Jean M. Arseneau‐Robar:** Formal analysis (equal); Writing‐original draft (supporting); Writing‐review & editing (equal). **Tyler Ronald Bonnell:** Formal analysis (equal); Writing‐original draft (supporting); Writing‐review & editing (equal). **Samantha M. Stead:** Data curation (equal); Formal analysis (equal); Writing‐original draft (supporting); Writing‐review & editing (equal). **Julie Annette Teichroeb:** Conceptualization (equal); Data curation (equal); Formal analysis (equal); Funding acquisition (equal); Supervision (equal); Writing‐original draft (equal); Writing‐review & editing (equal).

## Supporting information

Supplementary Material

## Data Availability

All data is archived in the *Dryad* data repository, https://doi.org/10.5061/dryad.866t1g1px.
