# Supplementary material for: Temporal patterns in the social network of core units in Rwenzori Angolan colobus monkeys: Effects of food availability and interunit dispersal
Source: Ecol Evol. 2021 Mar 5;11(7):3251–63. doi: 10.1002/ece3.7274 (PMC8019045; doi:10.1002/ece3.7274)
Supplement: Supplementary file 1 — Supplementary Material [file ECE3-11-3251-s001.docx]

**Temporal patterns in the social network of core units in Rwenzori Angolan colobus monkeys: effects of food availability and inter-unit dispersal**

Frances V. Adams, T. Jean Arseneau-Robar, Tyler R. Bonnell, Samantha M. Stead & Julie A. Teichroeb

**Supplementary Material**

**Table S1**. Network analysis statistics for the 13 core units within the *Colobus angolensis ruwenzorii* study band at Nabugabo, Uganda comparing sampling periods 1 (Aug. 28, 2017 – Aug. 22, 2018) and 2 (Aug. 29, 18 – May13, 19), with the highest value in each column in bold and the lowest underlined.

|  | **Affinity** | | **Strength** | | **Eigenvector centrality** | | **Reach** | | **Clustering coefficient** | |
| --- | --- | --- | --- | --- | --- | --- | --- | --- | --- | --- |
| **Sampling period** | **1** | **2** | **1** | **2** | **1** | **2** | **1** | **2** | **1** | **2** |
| **Core unit**  Albizia | 0.63 | 0.63 | 0.75 | 0.69 | 0.34 | 0.31 | 0.47 | 0.44 | 0.47 | 0.3 |
| Antiaris | 0.64 | 0.64 | 0.48 | 0.61 | 0.22 | 0.28 | 0.31 | 0.39 | 0.48 | 0.31 |
| Brighia | 0.66 | 0.64 | 0.59 | 0.6 | 0.28 | 0.28 | 0.39 | 0.38 | 0.53 | 0.33 |
| Fagara | 0.61 | 0.59 | 0.48 | 0.59 | 0.21 | 0.24 | 0.29 | 0.35 | 0.46 | 0.29 |
| Funtumia | 0.64 | 0.63 | 0.61 | 0.66 | 0.29 | 0.3 | 0.39 | 0.42 | 0.5 | 0.31 |
| Liana | 0.64 | **0.66** | **0.76** | 0.67 | **0.35** | 0.32 | **0.48** | 0.44 | 0.49 | 0.34 |
| Lovoa | **0.67** | 0.64 | 0.63 | 0.71 | 0.3 | **0.33** | 0.42 | **0.45** | **0.54** | 0.31 |
| Maesopsis | 0.61 | 0.62 | 0.59 | 0.54 | 0.26 | 0.23 | 0.36 | 0.33 | 0.44 | 0.3 |
| Newtonia | 0.64 | 0.62 | 0.58 | 0.57 | 0.27 | 0.25 | 0.37 | 0.35 | 0.49 | 0.32 |
| Phoenix | 0.64 | 0.61 | 0.7 | **0.72** | 0.33 | 0.32 | 0.45 | 0.44 | 0.5 | 0.29 |
| Polysiasis | 0.63 | 0.64 | 0.71 | 0.69 | 0.33 | 0.32 | 0.45 | 0.44 | 0.47 | 0.32 |
| Pseudospondias | 0.61 | 0.61 | 0.58 | 0.58 | 0.25 | 0.25 | 0.35 | 0.36 | 0.44 | 0.31 |
| All-male unit | na | 0.65 | na | 0.27 | na | 0.13 | na | 0.18 | na | 0.33 |
| **Mean (SE)** | 0.63 (0.02) | 0.63 (0.02) | 0.62 (0.09) | 0.61 (0.12) | 0.29 (0.05) | 0.27 (0.06) | 0.39 (0.06) | 0.38 (0.07) | 0.48 (0.03) | 0.31 (0.02) |

**Choice of Window Size**

To determine the best window size for the data under consideration, the netTS package tutorials provide guidance on choosing a lower time scale for window-size using a bootstrap technique. This technique measures the similarity between the observed network in a window and networks created using bootstrap samples from within the window. This bootstrap procedure first randomly samples with replacement from the observed association data within a window, generates a network from this sample, and compares this network to the observed network. The idea here is that the similarity between bootstrapped networks and the observed network provides information about the similarity of the observed to a simulated network in which all interactions were observed (i.e., if perfect sampling was possible), equivalent to the traditional bootstrapping approach (Efron, 1992). To also help choose a window size, we estimated the variability in network density, as window sizes too large will reach asymptote (i.e., density of 1) and window sizes too small will have densities close to 0, resulting in lower variation (Caceres, Berger-Wolf, & Grossman, 2011). The optimal window size, in terms of maximizing variability in edge density, for our data was 31 days. This choice of window size also showed reasonable correlations between the bootstrapped and observed networks over our study period (Fig. S1).


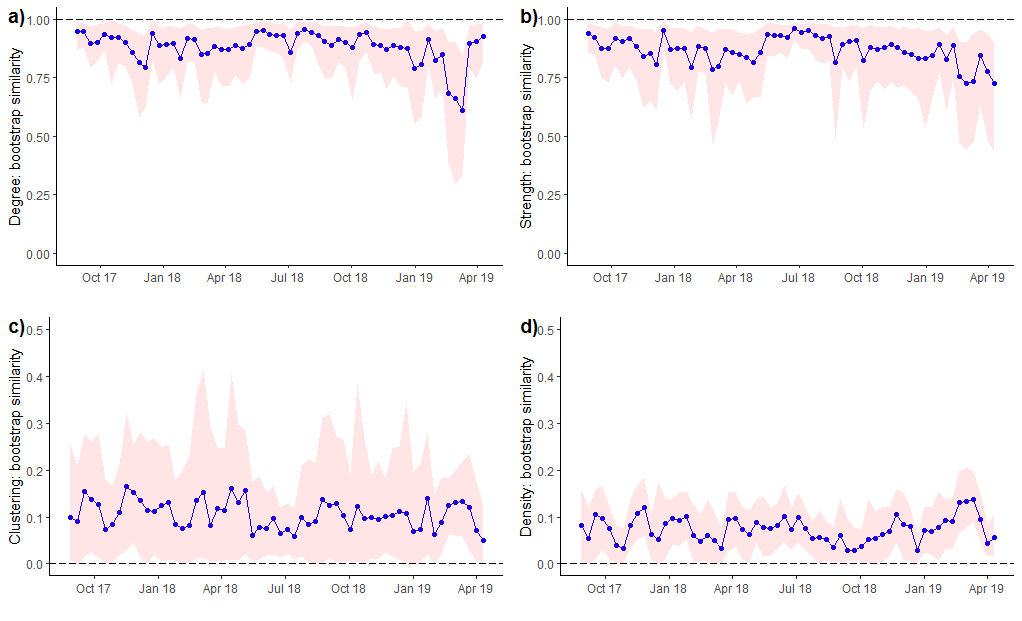


**Fig. S1.** Estimated similarity between the observed network measure and bootstrapped replicates in *Colobus angolensis ruwenzorii* core unit social networks at Nabugabo, Uganda: a) node degree, b) node strength, c) clustering coefficient, and d) edge density of the network. Similarity for node-level measures, a-b, is measured using Pearson’s correlation coefficient, while for global network measures, c-d, similarity is measured using Euclidean distance. The 95% CI, pink shaded areas, are generated from 1000 bootstrapped networks. Dashed lines indicate optimal outcomes, i.e., 1.0 for correlation similarity, and 0.0 for Euclidean distance.

**Results of Permutation Analysis on Regression Coefficients**

To help interpret the results of our network regressions we make use of a post-network permutation approach. Generally, the use of permutations with network data in linear regressions is used when a network measure (node/dyad) is used both as a response and a predictor variable (Weiss et al. 2020). As these measures potentially share some covariance, due to the fact they are measured on the same network, the use of permutations to randomly assign node/dyad weights can be very useful in interpreting regression coefficients independently of this shared covariance. In our regressions we only have network measures as response variables and environmental variables as predictor variables, and as such we do not have issues of potential shared covariance. We then use permutations to assess the chance that our observed coefficients could arise due to chance, and to match with current best practices in network analysis. Below we present the range of regression coefficient values fit to 100 permuted datasets, where the response variable was randomly permuted, thus keeping the same distribution as the observed data but where values are randomly assigned across core units and times. We present the 95% credible interval of the regression coefficients for the models fit to the permuted datasets, and compare that with the coefficients from the models fit to the observed data (Table S2). We also provide histograms to view the results graphically (Fig. S2).

**Table S2**. Estimated regression coefficients for the observed and 100 permuted datasets. To summarize the range of coefficient estimated from each of the 100 permuted datasets the 95% credible interval is presented for each model: core unit strength (weighted degree), core unit degree, network density of the full network, and clustering coefficient of the full network.

| **Model** | **Coefficient** | **Observed** | **Permutation 95%CI** |
| --- | --- | --- | --- |
| Strength | Fruit | 2.28 | -0.67, 0.58 |
|  | Leaves | -0.88 | -0.72, 0.66 |
|  | Rain | -0.83 | -0.64, 0.66 |
| Degree | Fruit | 0.84 | -0.16, 0.24 |
|  | Leaves | -0.23 | -0.19, 0.20 |
|  | Rain | -0.22 | -0.18, 0.22 |
| Density | Fruit | 0.08 | -0.04, 0.05 |
|  | Leaves | -0.02 | -0.04, 0.04 |
|  | Rain | -0.02 | -0.05, 0.04 |
| Clustering | Fruit | 0.07 | -0.04, 0.04 |
|  | Leaves | 0.00 | -0.05, 0.04 |
|  | Rain | 0.02 | -0.04, 0.04 |


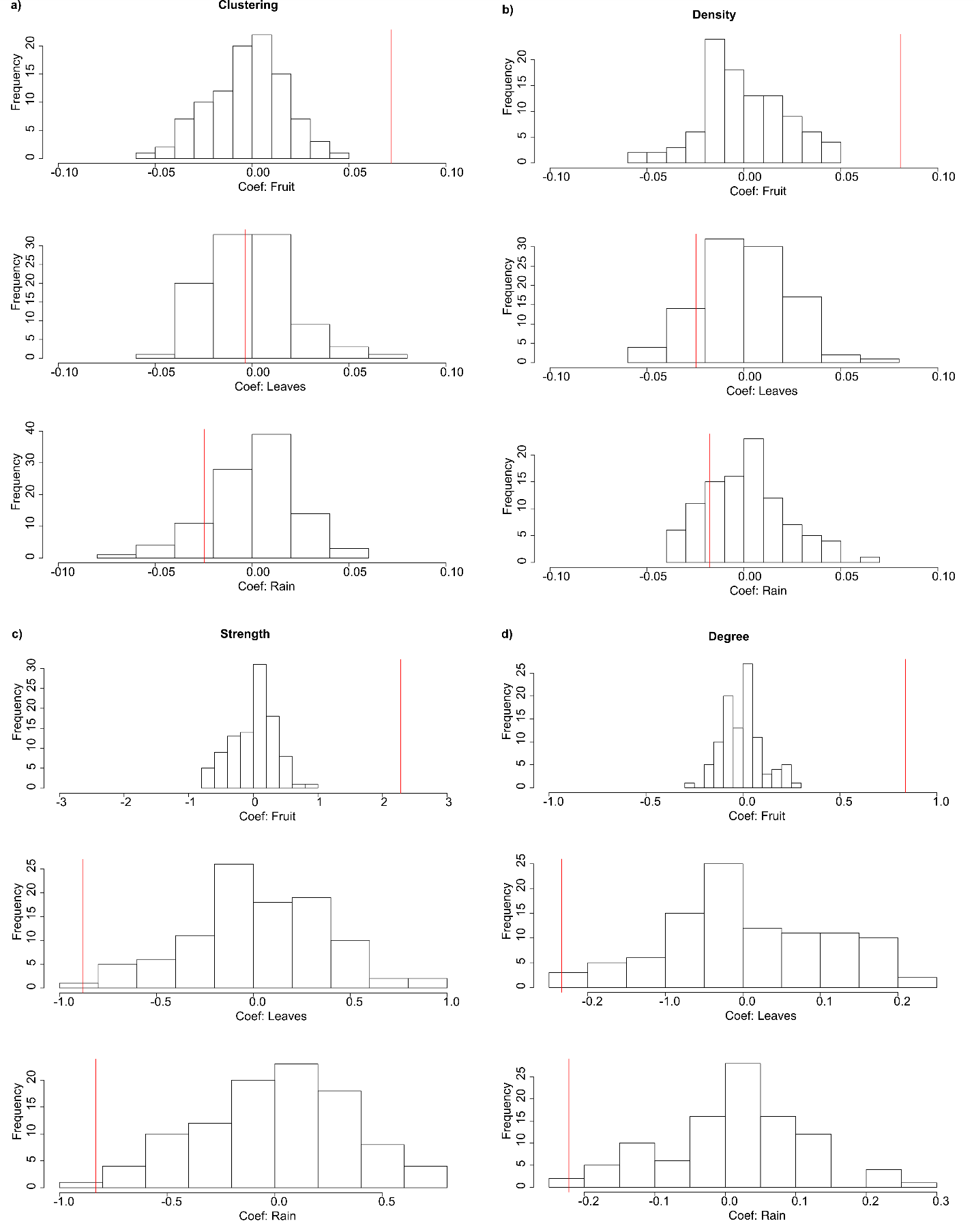


**Fig. S2**. Histograms of regression coefficients estimated with the permuted dataset, compared to the regression coefficient from the observed dataset.

*Posterior Predictive Checks*


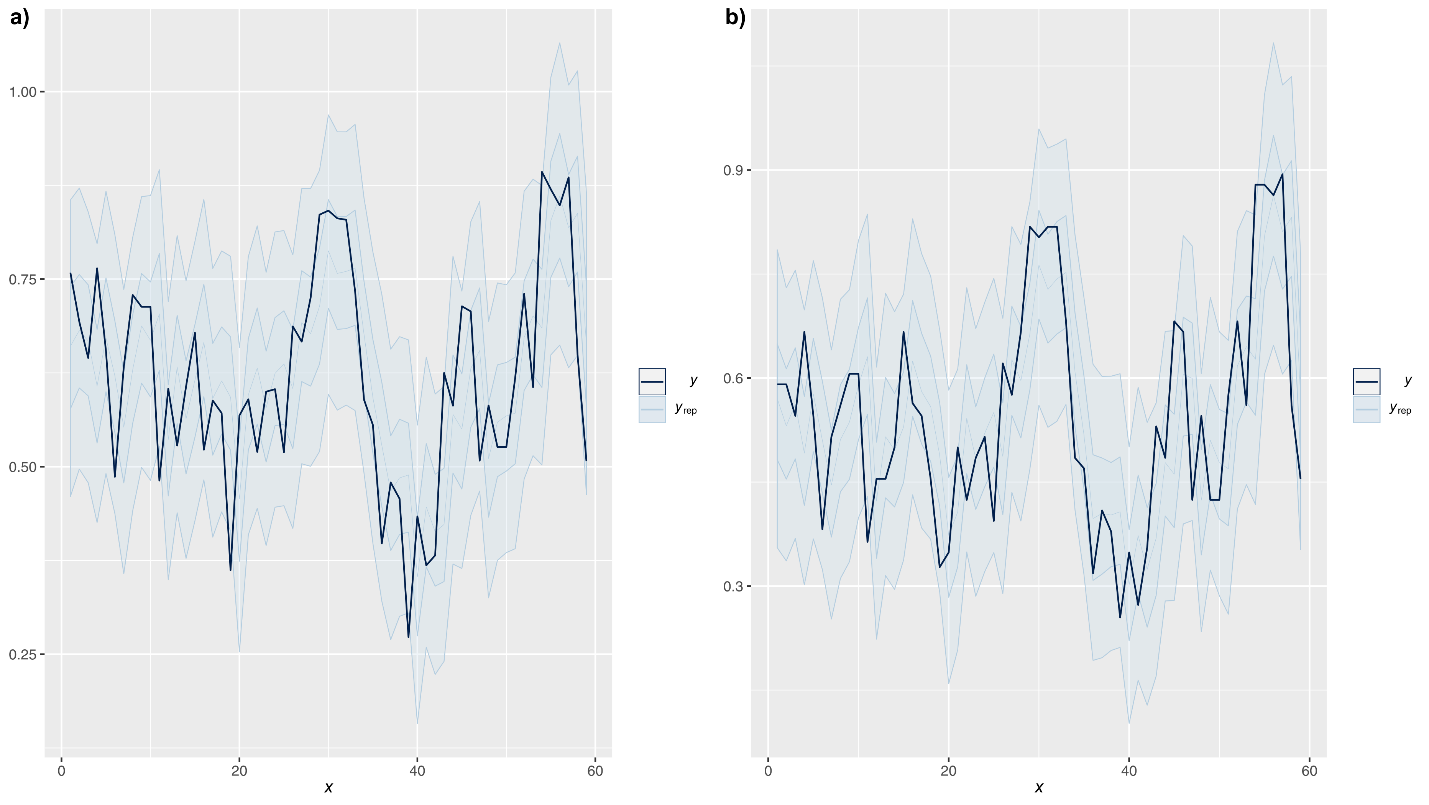


**Fig. S3**. Posterior predictive checks (ribbon plots) for the linear models with the response being: a) network density, and b) network clustering coefficient over time. Network predicted values (y_rep) are presented with the observed values (y). The x-axis is the order in time of the observed values.


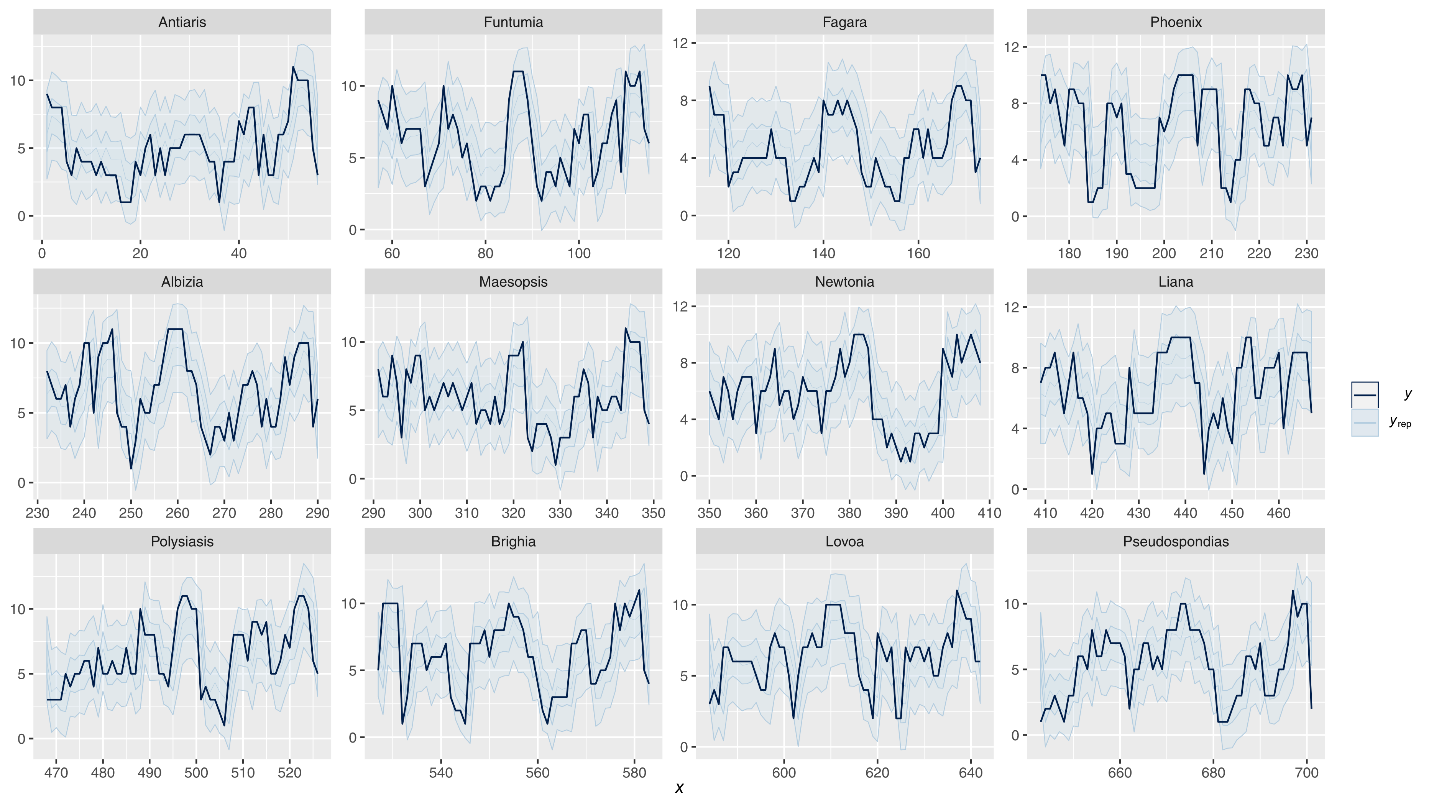


**Fig. S4**. Posterior predictive checks (ribbon plots) for the multilevel model with the response being core unit degree over time. Each individual’s predicted values (y_rep) are presented with the observed values (y). The x-axis is the order in time of the observed values.


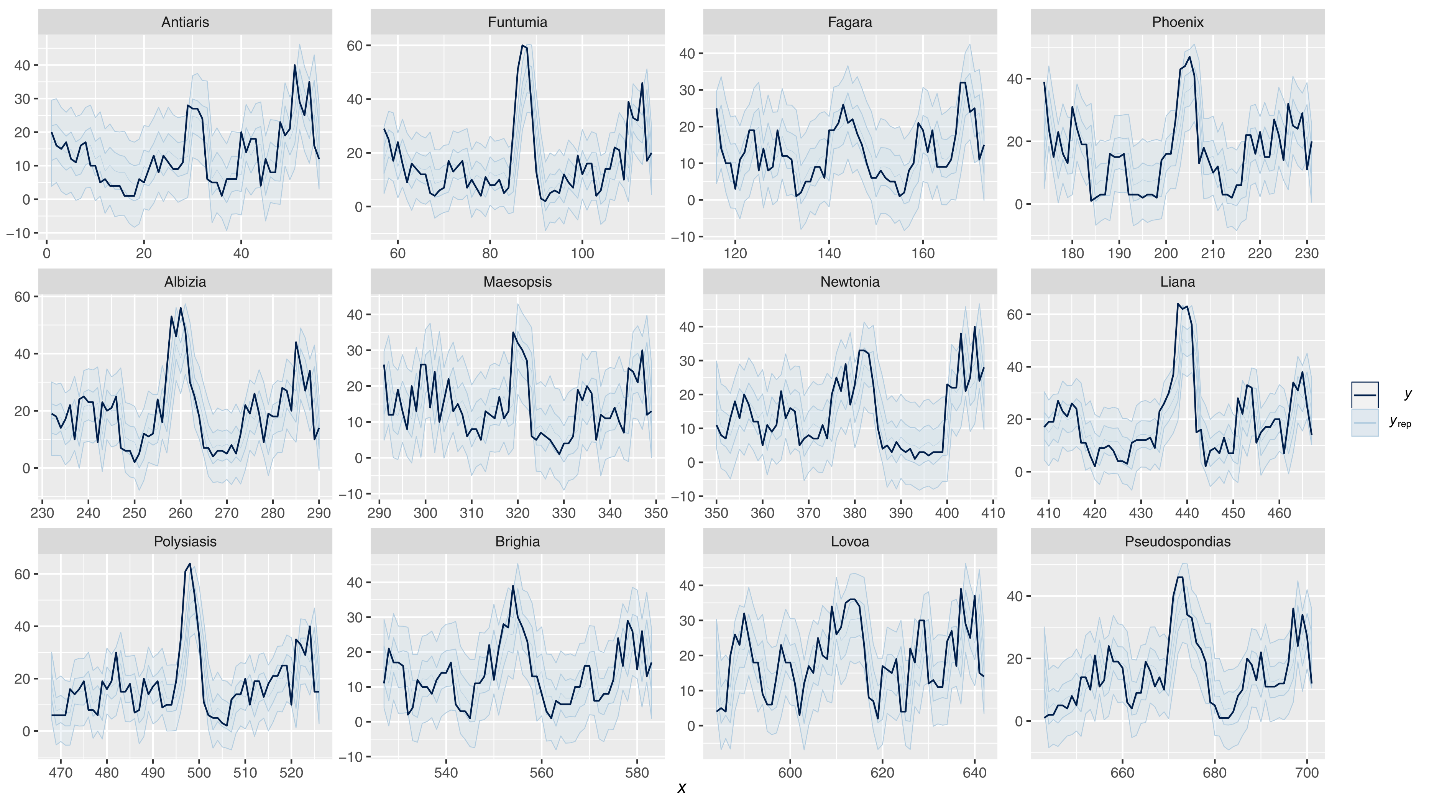


**Fig. S5**. Posterior predictive checks (ribbon plots) for the multilevel model with the response being core unit strength over time. Each individual’s predicted values (y_rep) are presented with the observed values (y). The x-axis is the order in time of the observed values.

**References**

Caceres, R.S., Berger-Wolf, T. & Grossman, R. (2011) Temporal scale of processes in dynamic networks. *Data Mining Workshops (ICDMW), 2011 IEEE 11th International Conference on*, pp. 925-932. IEEE.

Efron, B. (1992) Bootstrap methods: another look at the jackknife. In S. Kotz, & N. L. Johnson (Eds.), *Breakthroughs in statistics* (pp. 569-593). New York, NY: Springer-Verlag.

Weiss, M.N., Franks, D.W., Brent, L.J.N., Ellis, S., Silk, M.J. & Croft, D.P. (2020) Common datastream permutations of animal social network data are not appropriate for hypothesis testing using regression models. Methods in Ecology and Evolution 00, 1-11. doi: 10.1111/2041-210X.13508
